# Synthesis and Antibacterial Activities of 1-Alkyl-3-methacryloyl (Acryloyl) of Benzimidazolone (Thione) Derivatives

**DOI:** 10.3390/ijms13044819

**Published:** 2012-04-16

**Authors:** Shaopeng Wei, Wenjun Wu, Zhiqin Ji

**Affiliations:** College of plant protection and Institute of Pesticide Science, Northwest A & F University, Shaanxi 712100, China; E-Mails: weishaopeng8888@163.com (S.W.); wuwenjun@nwsuaf.edu.cn (W.W.)

**Keywords:** benzimidazolone (thione) derivatives, synthesis, antibacterial activity

## Abstract

A series of (28) 1-alkyl-3-methacryloyl (acryloyl)-benzimidazolone (thione) deriv-atives were synthesized. The structures of the new derivatives were confirmed by ^1^H-NMR, ^13^C-NMR and ESI-MS spectral analysis. The antibacterial activities of these compounds against several strains of bacteria, such as *Bacillus cereus*, *Bacillus subtilis*, *Escherichia coli*, *Staphylococcus aureus* and *Pseudomonas aeruginosa*, were evaluated by methods of paper disc-diffusion and broth mciro-dilution. Methacryloyl derivatives displayed higher antibacterial activities against tested bacterial strains than those of acryloyl derivatives in *in vitro* tests. A structure-activity relationship (SAR) study revealed that the presences of the methacryloyl moieties is essential to the antibacterial activities of the compounds.

## 1. Introduction

With the widespread use of antibiotics, the emergence of resistant bacteria has become a great threat to public health [1]. Although there are many approaches to limit the spread of resistant bacteria, the development of new antibiotics is undoubtedly a fundamental solution [2]. It is regretful that the number of newly approved antibiotics continues to decline, and the majority of compounds under different stages are improved variants of marketed antibiotics [3]. Finding good initial lead compounds and progressing compounds into the clinic has proven difficult owing to bottlenecks in the discovery pipeline [4]. Benzimidazolone derivatives have been found to exhibit anti-HIV [5], antitrichinellosis [6], antinociceptive [7], antitumor activities [8], and other pharmacological activities [9–12]. For instance, it has been reported in recent literature that benzimidazolone bearing a sugar or piperidine residue on the aromatic nitrogen effectively inhibits the growth of bacteria [13,14].

In previous studies, we synthesized a series of 1-acyl-3-isopropenyl-benzimidazol-2-ones, in which several derivatives of aromatic and α, β-unsaturated carboxylic acids showed strong activities against *Botrytis cinerea*, a plant pathogenic fungus that causes an important disease affecting vegetables and fruits [15]. In further investigation, their antibacterial activities against several strains of bacteria were assessed by broth micro-dilution method. The results showed that only 1-methacryloyl-3-isopropenyl-benzimidazol- 2-one exhibited extraordinary antibacterial activity, whereas other derivatives only showed minor or even no inhibition. A structure-activity relationship (SAR) study revealed that the presence of α, β-unsaturated carbonyl moiety is essential to the antibacterial activities of these derivatives. To better understand the SAR, a series of (28) 1-alkyl-3-methacryloyl (acryloyl)-benzimidazolone (thione) derivatives were synthesized and their antibacterial activities were evaluated herein.

## 2. Results and Discussion

### 2.1. Synthesis of 1-Alkyl-3-methacryloyl (Acryloyl)-benzimidazolone (Thione)

**Scheme 1:**
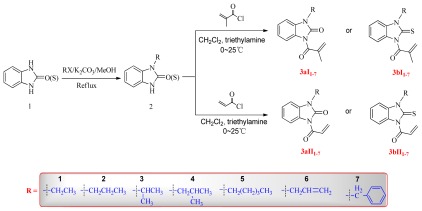
Synthetic route of the title compounds.

For the synthesis of the title compounds, the reaction sequences outlined in Scheme 1 were followed, starting from 2(3*H*)-benzimidazolone (thione) (**1**), which was prepared by the reaction of *O*-phenylenediamine and urea or thiourea. Compound **1** was reacted with corresponding alkylhalide to obtain 1-alkyl-2(3*H*)-benzimidazolone (thione) (**2**), and the title compounds were then obtained by the reaction of 1-alkyl-2(3*H*)-benzimidazolone (thione) with corresponding acid chloride, which was prepared by the reaction of the carboxylic acids with thionyl chloride [16]. The chemical structures of the compounds have been elucidated by ^1^H-NMR, ^13^C-NMR and ESI-HRMS spectral analysis.

### 2.2. Antibacterial Activities

Antibacterial activities of the title compounds against standard bacterial strains were evaluated by the method of paper disc-diffusion, and the results are listed in Table 1. MICs of 1-alkyl-3-meth-acryloyl-*Int.* benzimidazolone (thione) derivatives against tested bacterial strains were evaluated by the broth micro-dilution method, and the results are listed in Table 2.

As shown in Table 1, except for benzyl (**3aI_7_** and **3bI_7_**), most other alkyl derivatives of methacryloyl benzimidazolone (thione) (**3aI_1_~3aI_6_** and **3bI_1_~3bI_6_**) showed extraordinary antibacterial activity against both gram-positive and gram-negative bacteria, whereas acryloyl derivatives exhibited weak or even no active against tested bacteria. This suggests that the presence of α, β-unsaturated carbonyl is essential to the antibacterial activities, and the methyl group substituted at α-position is also important. The loss of activity of benzyl derivatives (**3aI_7_** and **3bI_7_**) may be due to the steric hindrance of benzene ring. In Table 2, the activities of pentyl derivatives (**3aI_5_** and **3bI_5_**) decreased dramatically compared to those of other alkylated derivatives, which implies that a longer carbon chain is not favorable to the activity. Obviously, the introduction of different alkyl groups to the N_1_-position of the moiety of benzimidazolone had various affect on the activity, and 2–4 carbon substitutions may be more favorable for promoting the antibacterial activity as compared with much larger substitution groups at the same position. The antibacterial activities of 2-hydroxyl and 2-hydrosulfuryl benzimidazoles were almost identical, which indicates that these two groups had similar biological effects. These results have encouraged us to start further investigations into novel benzimidazolone (thione) derivatives as antibacterial agents and this will be reported in due course.

## 3. Experimental Section

### 3.1. General Experimental Procedures

Melting points were measured on a WPR apparatus and are uncorrected (Shanghai Jingke Co., China). The ^1^H-NMR (500 MHz), and ^13^C-NMR (125 MHz) were obtained on a Bruker AM-500 FT-NMR spectrometer with CDCl_3_ as the solvent and TMS as the internal standard. MS were recorded under ESI conditions using an APEX II FT-ICR mass spectrometer (Bruker Daltonics Inc.). Analytical thin layer chromatography (TLC) was carried out on precoated plates (silica gel), and spots were visualized with H_2_SO_4_/EtOH.

### 3.2. Synthetic Procedures

#### 3.2.1. General Procedures for 1-Alkyl-Benzimidazolone (Thione) (**2**)

To a solution of **1** (1 mmol) in methanol (10 mL) were added potassium carbonate (0.21 g, 1.5 mmol) and alkylhalide (1 mmol), and the mixture was refluxed for 4 h. The reaction was cooled and evaporated to dryness in vacuum. The residue was dissolved in 10 mL of water and 20 mL of ethyl acetate, and then stirred for 10 min. The organic layer was separated and washed with water followed by brine. The aqueous layer was extracted twice with ethyl acetate. The combined organic layers were dried over Na_2_SO_4_, concentrated under vacuum and the crude product was purified by flash chromatography on silica gel.

#### 3.2.2. General Procedures for 1-Alkyl-3-Methacryloyl (Acryloyl)-Benzimidazolone (Thione) (**3**)

With nitrogen protection, to a solution of **2** (1 mmol) in anhydrous dichloromethane (10 mL) was added triethylamine (0.3 mL, 2.1 mmol). Carboxylic chloride (1 mmol) in dichloromethane (2 mL) was added in an ice-water bath, and the mixture was stirred for 5 h at room temperature. Saturated NaHCO_3_ solution (10 mL) was added, and the mixture was extracted by dichloromethane (3 × 15 mL). The organic extracts were combined, washed with water and saturated NaCl solution in that order, dried over Na_2_SO_4_, and finally concentrated under vacuum. After purification on a silica gel column, target compounds were obtained.

*1-ethyl-3-methacryloyl-1*H*-benzo[d]imidazol-2(3*H*)-one* (**3aI_1_**): white crystalline solid (0.145 g, 63% yield), mp 60–62 °C. ^1^H-NMR (500 MHz, CDCl_3_) *δ* 1.36 (t, *J* = 7.0 Hz, 3H), 2.15 (s, 3H), 3.91 (q, *J* = 7.0 Hz, 2H), 5.54 (s, 1H), 5.55 (s, 1H), 7.01 (d, *J* = 8.0 Hz, 1H), 7.16 (t, *J* = 8.0 Hz, 1H), 7.22–7.26 (m, 1H), 7.97 (d, *J* = 8.0 Hz, 1H); ^13^C-NMR (125 MHz, CDCl_3_) *δ* 13.1, 19.0, 36.0, 107.7, 115.0, 121.2, 122.4, 124.5, 126.6, 129.9, 140.7, 151.2, 170.4. ESI-MS: 231.1153 ([*M*+H]^+^, [C_13_H_14_N_2_O_2_+H]^+^; calc. 231.1134).

*1-propyl-3-methacryloyl-1*H*-benzo[d]imidazol-2(3*H*)-one* (**3aI_2_**): white crystalline solid (0.136 g, 56% yield), mp 67–68 °C. ^1^H-NMR (500 MHz, CDCl_3_) *δ* 0.99 (t, *J* = 7.0 Hz, 3H), 1.76–1.81 (m, 2H), 2.14 (s, 3H), 3.81 (t, *J* = 7.0 Hz, 2H), 5.53 (s, 1H), 5.55 (s, 1H), 7.00 (d, *J* = 8.0 Hz, 1H), 7.15 (t, *J* = 8.0 Hz, 1H),7.21–7.26 (m, 1H), 7.96 (d, *J* = 8.0 Hz, 1H); ^13^C-NMR (125 MHz, CDCl_3_) *δ* 11.3, 18.9, 21.3, 42.8, 107.8, 114.9, 121.2, 122.4, 124.4, 126.5, 130.4, 140.7, 151.6, 170.4. ESI-MS: 245.1295 ([*M*+H]^+^, [C_14_H_16_N_2_O_2_+H]^+^; calc. 245.1290).

*1-isopropyl-3-methacryloyl-1*H*-benzo[d]imidazol-2(3*H*)-one* (**3aI_3_**): yellow oil (0.158 g, 65% yield). ^1^H-NMR (500 MHz, CDCl_3_) *δ* 1.53 (d, *J* = 6.0 Hz, 6H), 2.14 (s, 3H), 4.62–4.64 (m, 1H), 5.52(s, 1H), 5.54 (s, 1H), 7.11 (d, *J* = 8.0 Hz, 1H), 7.18–7.23 (m, 2H), 7.96 (d, *J* = 8.0 Hz, 1H); ^13^C-NMR (125 MHz, CDCl_3_) *δ* 18.9, 21.7 (2CH_3_), 45.4, 108.9, 114.8, 121.0, 122.7, 124.3, 126.6, 129.3, 140.9, 150.9, 170.4. ESI-MS: 245.1280 ([*M*+H]^+^, [C_14_H_16_N_2_O_2_+H]^+^; calc. 245.1290).

*1-isobutyl-3-methacryloyl-1*H*-benzo[d]imidazol-2(3*H*)-one* (**3aI_4_**): white solid (0.142 g, 55% yield), mp 55–56 °C. ^1^H-NMR (500 MHz, CDCl_3_) *δ* 0.98 (d, *J* = 5.0 Hz, 6H), 2.14 (s, 3H), 2.19–2.22 (m, 1H), 3.64 (d, *J* = 6.5, 2H), 5.53 (s, 1H), 5.55 (s, 1H), 6.98 (d, *J* = 8.0 Hz, 1H), 7.13–7.26 (m, 2H), 7.96 (d, *J* = 8.0 Hz, 1H); ^13^C-NMR (125 MHz, CDCl_3_) *δ* 18.9, 20.2 (2CH_3_), 27.6, 48.6, 108.1, 114.8, 121.3, 122.4, 124.4, 126.5, 130.7, 140.7, 151.8, 170.4. ESI-MS: 259.1455 ([*M*+H]^+^, [C_15_H_18_N_2_O_2_+H]^+^; calc. 259.1447).

*1-pentyl-3-methacryloyl-1*H*-benzo[d]imidazol-2(3*H*)-one* (**3aI_5_**): white solid (0.166 g, 61% yield), mp 72–73 °C. ^1^H-NMR (500 MHz, CDCl_3_) *δ* 0.90 (CH_3_, br.s, 3H), 1.36 (2CH_2_, br.s, 4H), 1.75–1.76 (m, 2H), 2.14 (s, 3H), 3.83 (t, *J* = 7.0 Hz, 2H), 5.54 (s, 1H), 5.55 (s, 1H), 6.98 (d, *J* = 8.0 Hz, 1H), 7.06–7.23 (m, 2H), 7.96 (d, *J* = 8.0 Hz, 1H); ^13^C-NMR (125 MHz, CDCl_3_) *δ* 13.9, 18.9, 22.3, 27.5, 28.9, 41.2, 107.8, 114.9, 121.2, 122.4, 124.4, 126.6, 130.3, 140.7, 151.5, 170.4. ESI-MS: 273.1629 ([*M*+H]^+^, [C_16_H_20_N_2_O_2_+H]^+^; calc. 273.1603).

*1-allyl-3-methacryloyl-1*H*-benzo[d]imidazol-2(3*H*)-one* (**3aI_6_**): white solid (0.140 g, 58% yield), mp 42–44 C. ^1^H-NMR (500 MHz, CDCl_3_) *δ* 2.14 (s, 3H), 4.46 (d, *J* = 5.5 Hz, 2H), 5.26–5.29 (m, *2*H), 5.54 (s, 1H), 5.56 (s, 1H), 5.85–5.92 (m, 1H), 6.98 (d, *J* = 8.0 Hz, 1H), 7.14–7.26 (m, 2H), 7.96 (d, *J* = 8.0 Hz, 1H); ^13^C-NMR (125 MHz, CDCl_3_) *δ* 18.9, 43.5, 108.4, 114.9, 118.4, 121.3, 122.6, 124.5, 126.5, 130.1, 131.0, 140.6, 151.3, 170.3. ESI-MS: 243.1139 ([*M*+H]^+^, [C_14_H_14_N_2_O_2_+H]^+^; calc. 243.1134).

*1-benzyl-3-methacryloyl-1*H*-benzo[d]imidazol-2(3*H*)-one* (**3aI_7_**): white solid (0.188 g, 64% yield), mp 112–114 °C. ^1^H-NMR (500 MHz, CDCl_3_) *δ* 2.14 (s, 3H), 5.04 (s, 2H), 5.54 (s, 1H), 5.56 (s, 1H), 6.89–6.92 (m, 1H), 7.14–7.16 (m, 2H), 7.24–7.34 (m, 5H), 8.22–8.25 (m, 1H); ^13^C-NMR (125 MHz, CDCl_3_) *δ* 18.9, 44.8, 108.3, 116.2, 122.7, 124.6, 126.5, 127.5(2C), 128.1, 129.2(2C), 129.9, 132.0, 135.2, 140.5, 151.7, 170.3. ESI-MS: 293.1289 ([*M*+H]^+^, [C_18_H_16_N_2_O_2_+H]^+^; calc. 293.1290).

*1-ethyl-3-acryloyl-1*H*-benzo[d]imidazol-2(3*H*)-one* (**3aII_1_**): white crystalline solid (0.117 g, 54% yield), mp 45–47 °C. ^1^H-NMR (500 MHz, CDCl_3_) *δ* 1.36 (t, *J* = 7.0 Hz, 3H), 3.92 (q, *J* = 7.0 Hz, 2H), 5.98 (d, *J* = 11.0 Hz, 1H), 6.67 (d, *J* = 17.0 Hz, 1H), 6.99 (d, *J* = 8.0 Hz, 1H), 7.14–7.26 (m, 2H), 7.79 (dd, *J* = 17.0 Hz, 11.0 Hz, 1H), 8.24 (d, *J* = 8.0 Hz, 1H); ^13^C-NMR (125 MHz, CDCl_3_) *δ* 13.1, 36.0, 107.5, 116.1, 122.7, 124.7, 126.6, 129.2, 129.7, 131.8, 151.9, 165.3. ESI-MS: 217.0980 ([*M*+H]^+^, [C_12_H_12_N_2_O_2_+H]^+^; calc. 217.0977).

*1-propyl-3-acryloyl-1*H*-benzo[d]imidazol-2(3*H*)-one* (**3aII_2_**): white solid (0.115 g, 50% yield), mp 66–67 °C. ^1^H-NMR (500 MHz, CDCl_3_) *δ* 1.00 (t, *J* = 7.0 Hz, 3H), 1.80 (q, *J* = 7.0 Hz, 2H), 3.83 (t, *J* = 7.0 Hz, 2H), 5.98 (d, *J* = 10.0 Hz, 1H), 6.67 (d, *J* = 17.0 Hz, 1H), 6.99 (d, *J* = 7.5 Hz, 1H), 7.14–7.26 (m, 2H), 7.79 (dd, *J* = 17.0 Hz, 10.0 Hz, 1H), 8.24 (d, *J* = 7.5 Hz, 1H); ^13^C NMR (125 MHz, CDCl_3_) *δ* 11.3, 21.2, 42.8, 107.7, 116.1, 122.7, 124.7, 126.5, 129.3, 130.2, 131.8, 152.3, 165.4. ESI-MS: 231.1139 ([*M*+H]^+^, [C_13_H_14_N_2_O_2_+H]^+^; calc. 231.1134).

*1-isopropyl-3-acryloyl-1*H*-benzo[d]imidazol-2(3*H*)-one* (**3aII_3_**): white solid (0.138 g, 60% yield), mp 93–95 °C. ^1^H-NMR (500 MHz, CDCl_3_) *δ* 1.56 (d, *J* = 5.0 Hz, 6H), 4.68–4.70 (m, 1H), 5.97 (d, *J* = 10.0 Hz, 1H), 6.65 (d, *J* = 16.5 Hz, 1H), 7.13–7.26 (m, 2H), 7.76 (dd, *J* = 16.5 Hz, 10.0 Hz, 1H), 8.25 (d, *J* = 7.5 Hz, 1H); ^13^C-NMR (125 MHz, CDCl_3_) *δ* 19.8, 45.4, 108.8, 116.1, 122.3, 124.4, 126.7, 129.1, 129.5, 131.6, 151.6, 165.5. ESI-MS: 231.1130 ([*M*+H]^+^, [C_13_H_14_N_2_O_2_+H]^+^; calc. 231.1134).

*1-isobutyl-3-acryloyl-1*H*-benzo[d]imidazol-2(3*H*)-one* (**3aII_4_**): white solid (0.151 g, 62% yield), mp 106–108 °C. ^1^H-NMR (500 MHz, CDCl_3_) *δ* 1.00 (d, *J* = 5.0 Hz, 6H), 2.21–2.23 (m, 1H), 3.66 (d, *J* = 6.0 Hz, 2H), 5.98 (d, *J* = 10.5 Hz, 1H), 6.67 (d, *J* = 17.0 Hz, 1H), 6.97 (d, *J* = 7.5 Hz, 1H), 7.14–7.26 (m, 2H), 7.79 (dd, *J* = 16.0 Hz, 11.0 Hz, 1H), 8.23 (d, *J* = 7.5 Hz, 1H); ^13^C-NMR (125 MHz, CDCl_3_) *δ* 20.2(2CH_3_), 27.6, 48.6, 107.9, 116.0, 122.6, 124.6, 126.5, 129.3, 130.5, 131.8, 152.5, 165.4. ESI-MS: 245.1291 ([*M*+H]^+^, [C_14_H_16_N_2_O_2_+H]^+^; calc. 245.1290).

*1-pentyl-3-acryloyl-1*H*-benzo[d]imidazol-2(3*H*)-one* (**3aII_5_**): white solid (0.163 g, 63% yield), mp 54–56 °C. ^1^H-NMR (500 MHz, CDCl_3_) *δ* 0.89 (t, *J* = 6.0 Hz, 3H), 1.34–1.36(m, 4H), 1.70–1.73 (m, 2H), 3.78 (t, *J* = 7.5 Hz, 2H), 5.93 (dd, *J* = 10.5, 1.5 Hz, 1H), 6.63 (dd, *J* = 1.5, 17.0 Hz, 1H), 6.91 (d, *J* = 8.0 Hz, 1H), 7.04–7.17 (m, 2H), 7.78 (dd, *J* = 10.5 Hz, 17.0 Hz, 1H), 8.15 (d, *J* = 8.0 Hz, 1H); ^13^C-NMR (125 MHz, CDCl_3_) *δ* 13.9, 22.3, 27.5, 28.9, 41.0, 107.5, 115.8, 122.4, 124.5, 126.4, 129.3, 130.0, 131.4, 152.0, 165.0. ESI-MS: 259.1450 ([*M*+H]^+^, [C_15_H_18_N_2_O_2_+H]^+^; calc. 259.1447).

*1-allyl-3-acryloyl-1*H*-benzo[d]imidazol-2(3*H*)-one* (**3aII_6_**): yellow oil (0.135 g, 59% yield). ^1^H-NMR (500 MHz, CDCl_3_) *δ* 4.49–4.50 (d, *J* = 4.0 Hz, 2H), 5.25–5.29 (m, 2H), 5.87–5.92 (m, 1H), 5.99 (d, *J* = 10.0 Hz, 1H), 6.68 (d, *J* = 17.0 Hz, 1H), 6.97 (d, *J* = 8.0 Hz, 1H), 7.14–7.26 (m, 2H), 7.79 (dd, *J* = 17.0 Hz, 10.0 Hz, 1H), 8.24 (d, *J* = 8.0 Hz, 1H); ^13^C-NMR (125 MHz, CDCl_3_) *δ* 43.4, 108.2, 116.0, 118.3, 122.9, 124.7, 126.5, 129.2, 129.9, 130.9, 132.0, 152.0, 165.3. ESI-MS: 229.0980 ([*M*+H]^+^, [C_13_H_12_N_2_O_2_+H]^+^; calc. 229.0977).

*1-benzyl-3-acryloyl-1*H*-benzo[d]imidazol-2(3*H*)-one* (**3aII_7_**): white solid (0.206 g, 74% yield), mp 118–119 °C. ^1^H-NMR (500 MHz, CDCl_3_) *δ* 5.05 (s, 2H), 6.01 (dd, *J* = 10.0, 1.5 Hz, 1H), 6.69 (dd, *J* = 17.5, 1.5 Hz, 1H), 6.89–6.91 (m, 1H), 7.13–7.16 (m,2H), 7.26–7.34 (m, 5H), 7.83 (dd, *J* = 10.0, 1.5 Hz, 1H), 8.23–8.25 (m, 1H). ^13^C-NMR (125 MHz, CDCl_3_) *δ* 44.9, 108.3, 116.1, 122.9, 124.8, 126.5, 127.5(2C), 128.1, 129.0(2C), 129.2, 129.9, 132.0, 135.2, 152.5, 165.3. ESI-MS: 279.1133 ([*M*+H]^+^, [C_17_H_14_N_2_O_2_+H]^+^; calc. 279.1134).

*1-ethyl-3-methacryloyl-1*H*-benzo[d]imidazol-2(3*H*)-thione* (**3bI_1_**): white solid (0.128 g, 52% yield), mp 38–39 °C. ^1^H-NMR (500 MHz, CDCl_3_) *δ* 1.44 (t, *J* = 7.5 Hz, 3H), 2.19 (s, 3H), 3.33 (q, *J* = 7.5 Hz, 2H), 5.70 (s, 1H), 5.82 (s, 1H), 7.20–7.29 (m, 2H), 7.59 (d, *J* = 8.0 Hz, 1H), 7.63 (d, *J* = 8.0 Hz, 1H); ^13^C-NMR (125 MHz, CDCl_3_) *δ* 14.0, 18.6, 27.4, 113.4, 118.5, 123.2, 124.3, 126.1, 134.1, 139.3, 143.9, 154.4, 169.1. ESI-MS: 247.0900 ([*M*+H]^+^, [C_13_H_14_N_2_OS+H]^+^; calc. 247.0905).

*1-propyl-3-methacryloyl-1*H*-benzo[d]imidazol-2(3*H*)-thione* (**3bI_2_**): yellow oil (0.158 g, 61% yield). ^1^H-NMR (500 MHz, CDCl_3_) *δ* 1.07 (t, *J* = 7.0 Hz, 3H), 1.81 (q, *J* = 7.0 Hz, 2H), 2.18 (s, 3H), 3.30 (t, *J* = 7.0 Hz, 2H), 5.68 (s, 1H), 5.80 (s, 1H), 7.18 (t, *J* = 7.5, 1H), 7.25 (t, *J* = 7.5, 1H), 7.57 (d, *J* = 7.5 Hz, 1H), 7.61 (d, *J* = 7.5 Hz, 1H); ^13^C-NMR (125 MHz, CDCl_3_) *δ* 13.5, 18.5, 22.2, 35.0, 113.4, 118.5, 123.2, 124.2, 126.1, 134.1, 139.3, 143.9, 154.5, 169.0. ESI-MS: 261.1063 ([*M*+H]^+^, [C_14_H_16_N_2_OS+H]^+^; calc. 261.1062).

*1-isopropyl-3-methacryloyl-1*H*-benzo[d]imidazol-2(3*H*)-thione* (**3bI_3_**): yellow oil (0.148 g, 57% yield). ^1^H-NMR (500 MHz, CDCl_3_) *δ* 1.46 (d, *J* = 6.5 Hz, 6H), 2.17 (s, 3H), 4.13–4.15 (m, 1H), 5.66 (s, 1H), 5.79 (s, 1H), 7.18–7.27 (m, 2H), 7.57 (d, *J* = 8.0 Hz, 1H), 7.62 (d, *J* = 8.0 Hz, 1H); ^13^C-NMR (125 MHz, CDCl_3_) *δ* 18.5, 23.0(2CH_3_), 39.1, 113.3, 118.5, 123.3, 124.2, 126.3, 133.9, 139.4, 143.9, 153.8, 169.1. ESI-MS: 261.1063 ([*M*+H]^+^, [C_14_H_16_N_2_OS+H]^+^; calc. 261.1062).

*1-isobutyl-3-methacryloyl-1*H*-benzo[d]imidazol-2(3*H*)-thione* (**3bI_4_**): white solid (0.176 g, 64% yield), mp 55–57 °C. ^1^H-NMR (500 MHz, CDCl_3_) *δ* 1.07 (d, *J* = 5.0 Hz, 6H), 2.04–2.06 (m, 1H), 2.19 (s, 3H), 3.24 (d, *J* = 5.0 Hz, 2H), 5.69 (s, 1H), 5.82 (s, 1H), 7.18–7.28 (m, 2H), 7.57 (d, *J* = 8.0 Hz, 1H), 7.62 (d, *J* = 8.0 Hz, 1H); ^13^C-NMR (125 MHz, CDCl_3_) *δ* 18.5, 22.1, 28.1, 41.6, 113.3, 118.5, 123.2, 124.2, 126.2, 134.2, 139.3, 143.8, 154.8, 169.1. ESI-MS: 275.1213 ([*M*+H]^+^, [C_15_H_18_N_2_OS+H]^+^; calc. 275.1218).

*1-pentyl-3-methacryloyl-1*H*-benzo[d]imidazol-2(3*H*)-thione* (**3bI_5_**): yellow oil (0.196 g, 68% yield). ^1^H-NMR (500 MHz, CDCl_3_) *δ* 0.91 (d, *J* = 7.5 Hz, 3H), 1.33–1.47 (m, 4H), 1.74–1.78 (m, 2H), 2.16 (s, 3H), 3.31 (t, *J* = 7.5 Hz, 2H), 5.66 (s, 1H), 5.77 (s, 1H), 7.14–7.25 (m, 2H), 7.55 (d, *J* = 8.0 Hz, 1H), 7.60 (d, *J* = 8.0 Hz, 1H); ^13^C-NMR (125 MHz, CDCl_3_) *δ* 13.9, 18.5, 22.2, 28.4, 31.0, 33.0, 113.4, 118.5, 123.1, 124.2, 126.0, 134.1, 139.2, 143.9, 154.5, 168.9. ESI-MS: 289.1373 ([*M*+H]^+^, [C_16_H_20_N_2_OS+H]^+^; calc. 289.1375).

*1-allyl-3-methacryloyl-1*H*-benzo[d]imidazol-2(3*H*)-thione* (**3bI_6_**): colorless oil (0.137g, 53% yield). ^1^H-NMR (500 MHz, CDCl_3_) *δ* 2.16 (s, 3H), 3.97 (d, *J* = 7.5 Hz, 2H), 5.16 (d, *J* = 10.5, 1H), 5.35 (dd, *J* = 17.0, 1.0 Hz, 1H), 5.67 (s, 1H), 5.78 (s, 1H), 5.98–6.06 (m, 1H), 7.16–7.26 (m, 2H), 7.57 (d, *J* = 8.0 Hz, 1H), 7.60 (d, *J* = 8.0 Hz, 1H); ^13^C-NMR (125 MHz, CDCl_3_) *δ* 13.9, 18.5, 22.2, 28.4, 31.0, 33.0, 113.4, 118.5, 123.1, 124.2, 126.0, 134.1, 139.2, 143.9, 154.5, 168.9. ESI-MS: 259.0503 ([*M*+H]^+^, [C_14_H_14_N_2_OS+H]^+^; calc. 259.0509).

*1-benzyl-3-methacryloyl-1*H*-benzo[d]imidazol-2(3*H*)-thione* (**3bI_7_**): colorless oil (0.215g, 69% yield). ^1^H-NMR (500 MHz, CDCl_3_) *δ* 2.16 (s, 3H), 4.57 (s, 2H), 5.68 (s, 1H), 5.79 (s, 1H), 7.20–7.34 (m, 5H), 7.43–7.45 (m, 2H), 7.60 (d, *J* = 8.0, 1H), 7.66 (d, *J* = 8.0, 1H). ^13^C-NMR (125 MHz, CDCl_3_) *δ* 18.6, 37.8, 113.5, 118.6, 123.4, 124.4, 126.1, 127.7, 128.7(2C), 129.4(2C), 134.1, 136.2, 139.2, 143.8, 154.1, 168.9. ESI-MS: 309.1065 ([*M*+H]^+^, [C_18_H_16_N_2_OS+H]^+^; calc. 309.1062).

*1-ethyl-3-acryloyl-1*H*-benzo[d]imidazol-2(3*H*)-thione* (**3bII_1_**): white solid (0.132 g, 57% yield), mp 82–83 °C. ^1^H-NMR (500 MHz, CDCl_3_) *δ* 1.48 (t, *J* = 7.5 Hz, 3H), 3.36 (q, *J* = 7.5 Hz, 2H), 6.16 (d, *J* = 10.5 Hz, 1H), 6.73 (d, *J* = 17.0 Hz, 1H), 7.04–7.10 (dd, *J* = 17.0, 10.5 Hz, 1H), 7.23–7.33 (m, 2H), 7.63–7.66 (m, 2H),; ^13^C-NMR (125 MHz, CDCl_3_) *δ* 13.9, 27.0, 113.4, 118.8, 123.3, 124.6, 129.4, 133.2, 133.8, 144.2, 154.7, 164.4. ESI-MS: 233.0743 ([*M*+H]^+^, [C_12_H_12_N_2_OS+H]^+^; calc. 233.0749).

*1-propyl-3-acryloyl-1*H*-benzo[d]imidazol-2(3H)-thione* (**3bII_2_**): white solid (0.160 g, 65% yield), mp 100–102 °C. ^1^H-NMR (500 MHz, CDCl_3_) *δ* 1.08 (t, *J* = 7.5 Hz, 3H), 1.83–1.85 (m, 2H), 3.42 (q, *J* = 7.5 Hz, 2H), 6.16 (d, *J* = 10.5 Hz, 1H), 6.73 (d, *J* = 17.0 Hz, 1H), 7.07 (dd, *J* = 17.0, 10.5 Hz, 1H), 7.23–7.33 (m, 2H), 7.63–7.66 (m, 2H),; ^13^C-NMR (125 MHz, CDCl_3_) *δ* 13.4, 22.7, 38.8, 113.5, 118.7, 123.3, 124.6, 129.5, 133.3, 133.7, 144.1, 154.9, 164.4. ESI-MS: 247.0910 ([*M*+H]^+^, [C_13_H_14_N_2_OS+H]^+^; calc. 247.0905).

*1-isopropyl-3-acryloyl-1*H*-benzo[d]imidazol-2(3*H*)-thione* (**3bII_3_**): white solid (0.130 g, 53% yield), mp 102–103 °C. ^1^H-NMR (500 MHz, CDCl_3_) *δ* 1.51 (d, *J* = 6.0 Hz, 6H), 4.18–4.20 (m, 1H), 6.15 (d, *J* = 10.5, 1H), 6.72 (d, *J* = 17.0, 1H), 7.07 (dd, *J* = 17.0, 10.5 Hz, 1H), 7.23–7.26 (m, 1H), 7.29–7.32 (m, 1H), 7.64–7.66 (m, 2H); ^13^C-NMR (125 MHz, CDCl_3_) *δ* 23.0 (2CH_3_), 37.8, 113.5, 118.7, 123.3, 124.5, 129.6, 133.1, 133.6, 144.3, 154.1, 164.4. ESI-MS: 247.0910 ([*M*+H]^+^, [C_13_H_14_N_2_OS+H]^+^; calc. 247.0905).

*1-isobutyl-3-acryloyl-1*H*-benzo[d]imidazol-2(3*H*)-thione* (**3bII_4_**): colorless oil (0.153 g, 59% yield). ^1^H-NMR (500 MHz, CDCl_3_) *δ* 1.10 (d, *J* = 6.0 Hz, 6H), 2.08–2.10 (m, 1H), 3.28 (d, *J* = 6.0 Hz, 2H), 6.13 (d, *J* = 10.5, 1H), 6.69 (d, *J* = 17.0, 1H), 7.06 (dd, *J* = 17.0, 10.5 Hz, 1H), 7.21–7.28 (m, 2H), 7.59–7.62 (m, 2H); ^13^C-NMR (125 MHz, CDCl_3_) *δ* 22.2 (2CH_3_), 28.0, 41.1, 113.5, 118.7, 123.3, 124.5, 129.5, 133.3, 133.7, 144.1, 155.0, 164.4. ESI-MS: 261.1050 ([*M*+H]^+^, [C_14_H_16_N_2_OS+H]^+^; calc. 261.1062).

*1-pentyl-3-acryloyl-1*H*-benzo[d]imidazol-2(3*H*)-thione* (**3bII_5_**): colorless oil (0.186 g, 64% yield). ^1^H-NMR (500 MHz, CDCl_3_) *δ* 0.88 (t, *J* = 6.0 Hz, 3H), 1.35–1.38 (m, 4H), 1.77–1.80 (m, 2H), 3.53 (t, *J* = 7.5 Hz, 2H), 6.14 (d, *J* = 10.5), 6.70 (d, *J* = 17.0 Hz, 1H), 7.04 (dd, *J* = 17.0,10.5 Hz, 1H), 7.22–7.29 (m, 2H), 7.60–7.62 (m, 2H); ^13^C-NMR (125 MHz, CDCl_3_) *δ* 14.0, 22.2, 28.0, 29.0, 38.7, 113.6, 118.8, 123.3, 124.6, 129.4, 133.2, 133.7, 144.1, 152.0, 164.4. ESI-MS: 275.1211 ([*M*+H]^+^, [C_15_H_18_N_2_OS+H]^+^; calc. 275.1218).

*1-allyl-3-acryloyl-1*H*-benzo[d]imidazol-2(3*H*)-thione* (**3bII_6_**): white solid (0.161 g, 66% yield), mp 104–106 °C. ^1^H-NMR (500 MHz, CDCl_3_) *δ* 4.02 (d, *J* = 7.0 Hz, 2H), 5.15 (d, *J* = 10.0 Hz, 1H), 5.36 (dd, *J* = 16.5 Hz, 1H), 6.04–6.07 (m, 1H), 6.16 (d, *J* = 10.5, 1H), 6.72 (d, *J* = 17.0 Hz, 1H), 7.05 (dd, *J* = 17.0, 10.5 Hz, 1H), 7.22–7.29 (m, 2H), 7.60–7.62 (m, 2H); ^13^C-NMR (125 MHz, CDCl_3_) *δ* 35.5, 113.4, 119.1, 123.4, 124.6, 129.3, 132.6, 133.9, 143.5, 154.2, 164.3. ESI-MS: 245.0741 ([*M*+H]^+^, [C_13_H_12_N_2_OS+H]^+^; calc. 245.0749).

*1-benzyl-3-acryloyl-1*H*-benzo[d]imidazol-2(3*H*)-thione* (**3bII_7_**): colorless oil (0.223 g, 76% yield). ^1^H-NMR (500 MHz, CDCl_3_) *δ* 4.56 (s, 2H), 6.00 (dd, *J* = 10.0, 1.5 Hz, 1H), 6.68 (dd, *J* = 17.5, 1.5 Hz, 1H), 6.89–6.91 (m, 1H), 7.13–7.15 (m,2H), 7.27–7.34 (m, 5H), 7.83 (dd, *J* = 10.0, 1.5 Hz, 1H), 8.24–8.26(m, 1H). ^13^C-NMR (125 MHz, CDCl_3_) *δ* 37.9, 113.6, 119.1, 123.5, 124.7, 127.5(2C), 128.1, 129.1(2C), 129.3, 133.0, 133.9, 135.4, 143.8, 154.1, 164.5. ESI-MS: 295.0902 ([*M*+H]^+^, [C_17_H_14_N_2_OS+H]^+^; calc. 295.0905).

### 3.3. Antibacterial Activity

Antibacterial activities **3a(b)I_1–7_~3a(b)II_1–7_** were carried out by paper disc-diffusion method [17]. The standard bacterial strains *Bacillus cereus* (1.1846), *Bacillus subtilis* (1.88), *Staphylococcus aureus* (1.89), *Escherichia coli* (1.1574), and *Pseudomonas aeruginosa* (1.2031) were obtained from the China General Microbiological Culture Collection Center. Ampicillin sodium (Sigma, Shanghai, China) was used as positive control. Standardized inoculum (5 × 10^5^ cfu/mL) of each test bacterium was spread on to sterile Müller-Hinton agar (Hangzhou Microbial Reagent Co. Ltd., Zhejiang, China) plates so as to achieve a confluent growth. The title compounds were dissolved in dichloromethane at the concentration of 1 mg/mL, and then 5 μL of the solutions were transfered onto discs (diameter, 6 mm) punched from Whatman no. 1 filiter paper, so that the disc contained 5 μg of the compound. After the solvent was evaporated, the sample discs were placed gently on the previously-marked zones in the agar plates. Standard ampicillin (5 μg/disc) paper disc (diameter, 6 mm) was used as positive controls. The plates were allowed to stand for 1 h at 4 °C for diffusion to take place and then incubated at 37 °C for 24 h. The diameter of inhibition zone around each disc was measured and recorded at the end of the incubation period. Experiments were repeated triplicate and the average values are reported here.

### 3.4. Minimum Inhibitory Concentrations (MICs)

MICs of compounds **3a(b)I_1–6_** against five strains of bacteria were evaluated by the broth mciro- dilution method in 96-well plates [18]. The inoculum was prepared by suspending several colonies from an overnight culture of test bacteria from 0.5% sheep blood agar media in Müller-Hinton broth, and a adjusting to a 0.5 McFarland standard (approximately 1.5 × 10^8^ colony-forming units per mL). A further dilution of 1:200 was made by placing 0.25 mL of the adjusted suspension into 49.75 mL of Müller-Hinton broth. The compounds were firstly dissolved in DMSO at the concentration of 10 mg/mL, and it was diluted ten-fold with sterile water to give the stock solution. Two-fold serial dilutions of the tested compounds were prepared in Müller-Hinton broth. Then the dilutions and inoculated suspension of the bacteria were delivered to wells of a 96-well plate at the ratio of 1:1. The final concentration of inoculum in each well was 3.7 × 10^5^ colony-forming units per mL. After incubation for 24 h at 30 °C, the MICs were read. Experiments were repeated in triplicate and standard ampicillin was used as the positive control.

## 4. Conclusions

In summary, 28 new 1-alkyl-3-methacryloyl (acryloyl) derivatives of benzimidazolone (thione) (**3a(b)I_1–7_**~3**a(b)II_1–7_**) were synthesized and evaluated for their antibacterial activities against five standard bacterial strains *in vitro*. The bioassay of these analogues showed that all synthesized N_3_-methacryloyl-derivatives of benzimidazolone (thione) exhibited stronger active against tested bacterial strains, whereas the acryloyl-derivatives were weaker or inactive against tested bacterial strains. The data suggest that the substitutions of N_3_ have a notable influence on the antibacterial activity of the title compounds. In addition, 2–4 carbon substitutions may be more favorable for promoting the antibacterial activity as compared with much larger substitution groups at the N_1_ position. These results have encouraged us to start further investigations into novel benzimidazolone (thione) derivatives as antibacterial agents and this will be reported in due course.

## Figures and Tables

**Table 1:** Antibacterial activity of title compounds against five bacterial strains.

Compounds	Zone of inhibition (mm)				

	*B. cereus*	*B. subtilis*	*E. coli*	*S. aureus*	*P. aeruginosa*
**3aI** (1-alkyl-3-methacryloyl-benzimidazolone)					
1	14(+++)	13(+++)	19(+++)	11(++)	15(+++)
2	13(+++)	15(+++)	18(+++)	11(++)	14(+++)
3	15(+++)	15(+++)	19(+++)	-	20(+++)
4	13(+++)	14(+++)	17(+++)	-	16(+++)
5	10(++)	12(++)	14(++)	10(+)	9(++)
6	14(+++)	16(+++)	19(+++)	14(++)	16(+++)
7	-	-	-	-	-
**3aII** (1-alkyl-3-acryloyl-benzimidazolone)					
1	6(+)	7(+)	11(+)	-	-
2	7(+)	7(+)	-	-	-
3	7(+)	-	-	-	-
4	-	-	-	-	-
5	10	8	-	-	-
6	7	-	-	-	-
7	-	-	-	-	-
**3bI** (1-alkyl-3-methacryloyl-benzimidazolthione)					
1	13(+++)	13(+++)	15(+++)	13(++)	14(+++)
2	12(+++)	13(+++)	19(+++)	11(++)	14(+++)
3	14(+++)	14(+++)	18(+++)	9(+)	20(+++)
4	12(+++)	13(+++)	17(+++)	-	15(+++)
5	9(++)	8(+)	10(++)	9(+)	9(++)
6	12(+++)	11(+++)	15(+++)	12(+)	16(+++)
7	-	-	-	-	-
**3bII** (1-alkyl-3-acryloyl-benzimidazolthione)					
1	6(+)	6(+)	-	-	-
2	6(+)	-	12(+)	-	-
3	7(+)	-	-	-	-
4	-	-	-	-	-
5	-	-	-	-	-
6	7(+)	-	-	-	-
7	-	-	-	-	-
Ampicillin	15(+++)	16(+++)	18(+++)	13(++)	20(+++)

All values are means of three replicates; “-” means invisible “+” means eyeable, “++” means clear, “+++” means transparent. (Dose: 5 μg/disc).

**Table 2:** Minimum Inhibitory Concentrations (MICs) of **3aI** and **3bI** against five bacterial strains.

Compounds	MICs (μg/mL)				

	*B. cereus*	*B. subtilis*	*E. coli*	*S. aureus*	*P. aeruginosa*
**3aI** (1-alkyl-3-methacryloyl-benzimidazolone)					
1	6.25	3.13	3.13	25	25
2	12.5	1.56	6.25	50	25
3	3.13	3.13	1.56	>100	12.5
4	12.5	6.25	12.5	>100	50
5	100	100	>100	>100	>100
6	6.25	12.5	1.56	12.5	50
**3bI** (1-alkyl-3-methacryloyl-benzimidazolthione)					
1	12.5	6.25	12.5	50	25
2	12.5	3.13	6.25	50	25
3	3.13	6.25	3.13	>100	25
4	25	12.5	6.25	>100	50
5	>100	>100	>100	>100	>100
6	12.5	25	3.13	25	100
Ampicillin	6.25	1.56	3.13	6.25	25
